# Depression and HIV associated neurocognitive disorders among HIV infected adults in rural southwestern Uganda: a cross-sectional quantitative study

**DOI:** 10.1186/s12888-021-03316-w

**Published:** 2021-07-12

**Authors:** Jane Kasozi Namagga, Godfrey Zari Rukundo, Vallence Niyonzima, Joachim Voss

**Affiliations:** 1 Department of Nursing, Faculty of Medicine, Mbarara University of Science and Technology, Mbarara, Uganda; 2 Department of Psychiatry, Faculty of Medicine, Mbarara University of Science and Technology, Mbarara, Uganda; 3 Frances Payne Bolton School of Nursing, Case Western Reserve University, Cleveland, OH 44106 USA

**Keywords:** Beck depression inventory-1, Depression, HIV-associated neurocognitive disorder, International HIV dementia scale

## Abstract

**Background:**

HIV-Associated Neurocognitive Disorder (HAND remains a pronounced consequence of HIV/AIDS despite improved life expectancies. This is often associated with several dysfunctions such as decrease of attention, mood alterations and psychomotor disturbances. Many factors, including age, gender, employment status, and psychiatric disorders, have been associated with HAND. Among the associated psychiatric disorders, depression is often more prevalent. It can influence not only quality of life, relationships and employment but also adherence to medical care. We assessed the prevalence of depression and its association with HAND among people living with HIV in rural Southwestern Uganda.

**Methods:**

This was a cross-sectional study that used Beck Depression Inventory-1 and International HIV Dementia Scale to assess depression and HAND respectively. We defined depression with a score of > 10 and HAND with a cutoff score of ≤10. We conducted data analysis using STATA version 12, and Pearson Chi-square test and logistic regression to determine associations between depression and HAND. The level of statistical significance was set at *p* ≤ 0.05. Ethical approval and administrative clearance were obtained from relevant bodies.

**Results:**

Of the 393 participants assessed for depression and HAND, 27% had depression and 58.3% screened positive for HAND. All levels of depression were more prevalent among female participants. We found a significant association between depression and HIV associated neurocognitive disorders (χ2 (3) = 9.0538 *p* = 0.029).

**Conclusion:**

Our findings confirmed a high prevalence of depression in individuals with HAND which is a major component of the disease burden.

## Background

Worldwide, the increasing access and use of HIV treatment has brought a dramatic change in the demographics of people living with HIV (PLWH) [1]. This has increased their longevity [2]. HIV infection targets the central nervous system in subcortical brain areas, causing brain impairment [3]. This increases the patients’ risk for various neurocognitive disorders such as HIV-Associated Neurocognitive Disorders (HAND) [4]. HAND is defined as impairment of multiple cognitive domains in association with HIV in the absence of other causes [5, 6]. Many factors, including age, gender, employment status and psychiatric disorders, have been associated with HAND. Among psychiatric disorders, depression is often more prevalent [7]. According to International statistical classification of diseases and related health problems (ICD) & Diagnostic and Statistical Manual of Mental Disorders, Fifth Edition (DSM-5), depression is defined as a mood disorder characterized by persistently low mood, a feeling of sadness and hopelessness and loss of interest in activities they once enjoyed [8, 9].

Several adverse outcomes are associated with depression such as failure to adhere to recommended medical care, diet, exercise, impaired functional status, reduced quality of life, increased morbidity and mortality [7, 10–12]. However, insufficient attention has been paid to psychiatric conditions in HIV population in sub-Saharan Africa (SSA) notably in Uganda where most PLWH live and are in care [13].

Some studies in Zimbabwe and Kenya revealed that depression among PLWH is associated with poor health status, faster progression to AIDS and increased mortality [13, 14]. Furthermore, depression leads to reduced economic productivity and working abilities, social isolation and difficulties in problem solving [13]. According to Memiah, Shumba [15] and Wroe, Hedt-Gauthier [16], depression has been shown to predict non-adherence to ART.

A study by Nel and Kagee [17] in South Africa reported that non-adherent patients had a 3-fold higher risk of presenting moderate to severe depressive symptoms in comparison to adherent patients. The current study assessed the prevalence of depression and its association with HAND among HIV infected adults in rural southwestern Uganda.

## Materials and methods

### Study area

The study was conducted at The AIDS Support Organization (TASO) in Mbarara and Rukungiri districts in southwestern Uganda. TASO is the largest Non-Governmental Organization that provides comprehensive HIV/AIDS care that includes: HIV voluntary counseling and testing (VCT), antiretroviral therapy (ART) for adults and pediatric patients, prevention of maternal to child transmission (PMTCT), testing and routine laboratory services, including viral load testing. The TASO care management model is focused on individual patient, tailored to medication adherence, disease monitoring, and emphasizes home based care [18]. These services are delivered through community drug distribution points (CDDP) and /or at TASO centres and community outreaches.

### Study design

A cross-sectional study was conducted between April and July, 2017 among people living with HIV (PLWH) attending TASO centres in Southwestern Uganda. This provided a snapshot of the prevalence of depression and its association with HAND over a short period of time within these centres.

### Study procedures and data collection

The sample size was calculated by a mathematical expression, N = Z^2^PQ/D^2^ (Lwanga & Yook, 1986), where: N = Sample size**,** Z = Normal distribution at 1.96 that corresponds to 95% confidence interval**,** *P* = 50% (0.5) is the estimated proportion in the target population with depression**,** D = Margin of errors allowed which correspond to 0.05 error, Q = Estimated proportion without depression (1-p) = 50% (0.5).

*N* = 1.96^2^*0.5*0.5/0.05^2^ = 384.16**.** The calculated sample was 384 participants and we added 9 participants to compensate for any missing data. Our total sample was 393 participants.

We consecutively enrolled 393 participants on clinic days that willingly participated, giving a response rate of 100%. Recruitment was done by the first author (JKN) and trained research assistants (RAs). Prior to data collection, the RAs were trained by a psychiatrist to gain confidence and competence. Additionally the RAs were fluent in English and Runyakitara (the local language) understood by the participants. A written informed consent was obtained from the participants preceding to participation. Recruitment was pursued until the calculated sample size of 393 was reached. Permission to review the patients’ medical records was sought from the participants and directors of the study sites.

We included PLWH who were on ART and were between 18 and 50 years old. We excluded individuals who were above 50 years or older since old age is a known risk factor for cognitive impairments [19]. Evidence shows that the risk of experiencing depression increases with age due to some natural body changes such as folate deficiency [20]. Folic acid is important for the nervous system functioning and its deficiency affects mood and cognitive function, especially in older people. To ensure that there were no other obvious causes of depression other than HIV; we further excluded those individuals that had a history of depression and other chronic illnesses before HIV infection to minimize confounding factors. The functional impairment associated with medical illnesses often causes depression [21]. These included; 1) opportunistic infections of the central nervous system (CNS), 2) regular substance abuse, 3) a positive diagnosis of schizophrenia 4) thyroid dysfunction, 5) diabetes mellitus and/or hypertension, and 6) the deaf 7) mentally disabled and 8) pregnant women.

### Data collection instruments

The Beck Depression Inventory Scale I (BDI- I) and International HIV Dementia Scale (IHDS were used to screen depression and HAND among study participants respectively. Though, the tools have been tested and validated [20, 21], they are subjective scales used for screening purposes, which have to be further evaluated to confirm the diagnosis.

The Beck Depression Inventory (BDI) is a 21-item measure that is most widely used for screening the severity of depression [22]. The inventory is composed of items relating to depressive symptoms such as hopelessness and irritability, cognitions like guilt or feelings of being punished, as well as physical symptoms; fatigue, weight loss, and lack of interest in sex. Each item had four responses ranging in intensity from 0 to 3 giving a total possible score ranging from 0 to 63. This was computed and compared to the key scale to determine the severity of depression. A score of 0–9 suggested absence or minimal depressive symptoms whereas scores from 10 to 18, 19–29, and 30–63 were suggestive of the presence of mild or moderate, and severe depressive symptoms respectively. Therefore a score < 10 means no depression while a score ≥ 10 mean presence of depressive symptoms. The BDI-1 has been used in many studies to evaluate depression levels among different populations that include SSA, China, Portugal, and Brazil [23–26].

According to Beck, Ward [27] and Beck, Steer [28], BDI-1 was introduced in 1961 and since then its reliability and validity have been established across a broad spectrum of clinical and non-clinical populations.

The HIV associated neurocognitive disorder was screened using Sacktor’s IHDS [29]. This tool consists of three parts–motor speed, psychomotor speed, and memory. Each part assesses a specific cognitive domain and scores up to a maximum of four points. The final score is the sum of the three sub-scores with a range from 0 to12points. Motor speed was assessed through finger tapping, psychomotor speed through a defined alternating hand sequence, and memory recall through a 4 word recall after the first two assessments are performed. The IHDS has been validated for screening HAND in the United States, Uganda, Ethiopia, Argentina and South Africa [29, 30]. The tool has been recommended for use in research studies because it is easy to administer by all trained health workers. Another advantage of the IHDS is, that its administration requires no sophisticated instrumentation other than a watch with a second hand, and the instrument is independent of language and culture.

The BDI-1 and IHDS were translated into Runyakitara which is the predominant language spoken by the participants and back translated to English in order to maintain the original meaning. An additional socio-demographic questionnaire was designed to elicit demographic information including age, gender, education, occupation and other demographic variables.

### Statistical analysis

Data were entered and analyzed using STATA version 12 and presented using descriptive statistics. Chi square, Univariate and multivariate logistic regression analyses were performed to determine the association between depression and HAND with the level of statistical significance set at *p* ≤ 0.05.

## Results

Of the 393 that participated in the study, 105 (26.7%) were males and 288(73.3%) were females. The demographic characteristics of the participants are presented in Table 1 below:

**Table 1:** Association between demographic characteristics and depression

Demographic characteristic	Total cohort ***n*** = 393 (%)	Depression classification				Χ^**2**^	***P*** value
		Minimal n (%)	Mild n (%)	Moderate n(%)	Severe n (%)		
**Age**							
18–30	84 (21.4)	28 (68.2)	22 (25.9)	4 (4.7)	1 (1.2)	3.04	0.80
31–43	181 (46.1)	140 (76.9)	35 (19.2)	6 (3.3)	1 (0.6)		
44–50	128 (32.5)	90 (70.3)	32 (25.0)	5 (3.9)	1 (0.8)		
**Marital status**							
Single	49 (12.5)	32 (65.3)	15 (30.6)	1 (2.0)	1 (2.0)	28.7	0.018*
Married	186 (47.3)	148 (79.6)	33 (17.7)	4 (2.2)	1 (0.5)		
Divorced/separated	58 (14.8)	39 (67.2)	13 (22.4)	6 (10.3)	0 (0.0)		
Widow/widower	100 (25.4)	68 (68.0)	28 (28.0)	3 (3.0)	1 (1.0)		
**Education level**							
No formal	32 (8.1)	17 (53.1)	11 (34.4)	3 (9.4)	1 (3.1)	28.2	0.02*
Primary	221 (56.2)	163 (73.7)	48 (21.7)	9 (4.1)	1 (0.5)		
Secondary	101 (25.7)	79 (78.2)	19 (18.8)	3 (3.0)	0 (0)		
Vocational/Tertiary	39 (9.9)	28 (71.8)	10 (25.6)	0 (0.0)	1 (2.6)		
**Employment**							
Full time	129 (23.8)	102 (79.1)	22 (17.1)	5 (3.8)	0 (0.0)	23.0	0.34
Part time	55 (14.0)	40 (72.7)	10 (18.2)	3 (5.5)	2 (3.6)		
Peasant	178 (45.3)	122 (68.5)	49 (27.5)	6 (3.4)	1 (0.6)		
Unemployed	13 (3.3)	7 (53.8)	5 (38.5)	1 (7.7)	0 (0.0)		
Others	18 (4.6)	16 (88.9)	2 (11.1)	0 (0.0)	0 (0.0)		
**Religion**							
Anglican	212 (53.9)	151 (71.2)	49 (23.1)	10 (4.7)	2 (0.9)	6.4	0.70
Catholic	131 (33.3)	97 (74.0)	30 (22.9)	3 (2.3)	1 (0.8)		
Moslem	21 (5.3)	17 (81.0)	2 (9.5)	2 (9.5)	0 (0.0)		
Others	29 (7.4)	22 (75.9)	7 (24.1)	0 (0.0)	0 (0.0)		

*reflects statistically significant results (*P*< or = 0.05)

The Chi square analysis showed that education level (χ2 (15) = 28.2 *p* = 0.02) and marital status (χ2 (15) = 28.7 *p* = 0.018) were associated with depression respectively. Religion, age and employment were not associated with depression. One way ANOVA revealed that the divorced were more associated with depression (F (6, 388) = 2.82, *p* = .0108). Similarly, participants with secondary (*p* = 0.018) and tertiary education level (*p* = 0.042) were more associated with depression compared to those without formal education.

Table 2 shows the depression classification of the participants who were living with HIV.

**Table 2:** Classification of depression according to sex

Depression classification	Total cohort n (%)	Sex n (%)	
	393 (100)	Male 105 (26.7)	Female 288 (73.3)
Minimal	287 (73.0)	88 (22.4)	199 (50.6)
Mild	88 (22.4)	15 (3.8)	73 (18.6)
Moderate	15 (3.8)	1 (0.3)	14 (3.5)
Severe	3 (0.8)	1 (0.3)	2 (0.5)
Pearson chi square Value	*χ2* (3) = 9.63, *p* = 0.022		

The results show that there is a statistical significant difference between the four classifications of depression (*χ2* (3) = 9.63, *p* = 0.022). Majority (73.0%) of the participants had minimal depression and out of these, 22.4% were males and 50.6% females. The minority had moderate (0.3% males and 3.5% females) and severe depression (0.3% males and 0.5% females) respectively. The prevalence of depression was found to be 27%, higher in females (22.6%) than males (4.4%).

### Determinants of depression

Logistic regression analysis was conducted to determine the risk factors associated with depression (Table 3).

**Table 3:** Univariate and Multivariate logistic regression analysis for determinants of depression

Variable	Univariate analysis			Multivariate analysis		
	COR	95%CI	***P*** value	AOR	95%CI	***P*** value
**Age**						
18–30	Ref			Ref		
31–43	0.86	0.54–0.92	0.132	0.56	0.32–0.68	0.263
44–50	2.01	1.25–3.20	0.018*	1.86	1.36–2.06	0.023*
**Sex**						
Male	Ref			Ref		
Female	2.52	2.33–2.81	0.003*	2.01	1.76–2.51	0.005*
**Employment**						
Full time	Ref			Ref		
Part time	0.84	0.46–1.47	0.09	0.64	0.12–0.84	0.106
Peasant	0.54	0.23–1.16	0.12	0.44	0.014–0.83	0.16
Unemployed	1.76	1.12–2.56	0.004*	0.96	0.46–1.98	0.08
**Education Level**						
No formal education	Ref			Ref		
Primary	0.69	0.36–1.34	0.737	0.48	0.21–1.24	0.42
Secondary	1.034	1.015–2.06	0.04*	0.93	0.46–2.30	0.12
Vocational/Tertiary	1.46	1.024–2.98	0.003*	0.84	0.28–2.40	0.08
**Drug adverse effect**						
Present						
Absent	0.98	0.234–1.43	0.46			
**Treatment line**						
First line	Ref					
Second line	1.70	1.24–2.14	0.004*	1.08	0.98–1.94	0.069
**Regimen**						
Non EFV based	Ref					
EFV based	1.04	0.82–1.37	0.067			
ART duration	0.39	0.104–1.03	0.069			

*reflects statistically significant results (*P*< or = 0.05)

Univariate logistic regression showed that being a female (OR 2.52, *P* value 0.003), age between 44 and 50 (OR 2.01, *P* value 0.018), unemployed (OR 1.76, *P* value 0.004), secondary (OR 1.034, *P* value 0.04) and tertiary education level (OR 1.46, 0.003) and being on second line ART were associated with depression. Multivariate logistic regression showed that age between 44 and 50 (aOR 1.86, *p* value 0.023) and being a female (aOR 2.01 *P* value 0.005) were associated with depression.

### Association between HAND and depression

The association between HAND and depression are displayed below (Table 4).

**Table 4:** Association between depression and HAND

Depression classification	TOTAL COHORT ***n*** = 393 (%)	HAND ***n*** = 229 (%)	NO HAND ***n*** = 164 (%)	Pearson χ2
Minimal	287 (73.0)	156 (39.7)	131 (33.3)	χ2(3) = 9.0538 *p* = 0.029*
Mild	88 (22.4)	58 (14.8)	30 (7.6)	
Moderate	15 (3.8)	13 (3.3)	2 (0.5)	
Severe	3 (0.8)	2 (0.5)	1 (0.3)	

*reflects statistically significant results (*P*< or = 0.05)

The prevalence of HAND was 58.3%. Pearson Chi square test analysis showed that depression was significantly associated with HAND (χ2 (3) = 9.0538, *p* = 0.029).

## Discussion

We aimed to assess the prevalence of depression and its association with HAND among people living with HIV in rural Southwestern Uganda. We found depression prevalence of: 73% minimal, 22.4% mild, 3.8 moderate and 0.8% severe. In these depressive episodes, the patient suffers from lowering of mood, reduction of energy, and decrease in activity. There is lack of concentration with marked tiredness and reduced capacity for enjoyment and interest in any activity. Sleep is usually disturbed and appetite diminished. Usually, there is absence of self-confidence and self-esteem even in the mild form.

While the mild depressive episode presents with two or three of the above symptoms, the patient is usually distressed but will be able to continue with most activities. The moderate depressive episode presents with four or more of the above symptoms, the patient is likely to have great difficulty in continuing with ordinary activities. With severe depressive episode, several of the above symptoms are marked and distressing, typically loss of self-esteem and ideas of worthlessness or guilt [8].

In our current study, the overall prevalence of depression was found to be 27%. It was more prevalent in females (22.6%) than males (4.4%) and much lower compared to the findings of Kagee and Martin [31] that estimated a prevalence of moderate and severe depression of 37.4 and 20%, respectively. The lower rates may be to the continued use of ART which has improved the health of PLWH. Similarly, studies conducted in Brazil reported a depression prevalence of 32–34% [32, 33] which shows a great association with neurocognitive loss. Though the prevalence of depression in the current study was lower than in other studies in South Africa and Brazil, it could not be neglected. The results suggest that a considerable proportion of PLWH may be experiencing psychiatric difficulty, for which they may not be receiving treatment. Clarke and Currie [34] also asserted that chronic conditions such as HIV are a major risk factor for depression more so in individuals that may be cognitively impaired. Multivariate logistic regression showed age between 44 and 50 (aOR 1.86, *p* value 0.023) and being a female was 2 times more likely to be associated with depression (aOR 2.01 *P* value 0.005). This may probably be due to women being more likely to suffer a greater number of stressful life experiences compared with men. This is in line with the study that reported the prevalence of major depression being higher in women (5.5%) than in men (3.2%) in 2010, representing a 1.7-fold greater incidence in women [35, 36]. Studies from developed countries suggest that the differential risk may primarily stem from biological sex differences where estrogen present may be protective in males [37].

With less efficient immune system, older patients receiving ART are at an increased risk of neuropsychological impairment such as depression [38]. With aging, PLWH experience more complications in the physiological and psychological domains of which little attention is given [39]. In sub-Saharan Africa, up to 25% of PLWH suffer from some of depression [40] and some studies indicate that older age is associated with depressive symptoms coupled with HAND [41] . A recent study has also reported a 24% increased risk of depression in older PLWH [42] and are more likely to develop viral resistance because of low level of ART adherence.

Conversely, HAND is on increase despite the era of effective antiretroviral therapy. In the present study, out of 393 participants, 58.3% screened positive for HAND. This is in consistency with other studies that used similar tools to screen for HAND [29, 43]. The associated factors were gender, peasant farming and older age. This agrees with Cross, Önen [10] who reported that socio-demographic factors such as age, gender, occupation, marital status might influence the likelihood of developing HAND.

We found a significant association between depression and HAND. Depression can lead to neurocognitive deficits indirectly through interference with medication adherence. This is different from other studies which reported that neurocognitive impairment and depression are independent complications of HIV [44, 45]. This might be attributed to the fact that most of the participants had advanced HIV disease due to delayed treatment initiation.

Despite the strong link between depression and HAND, it is striking that there is no attention given to depression in the routine HIV care yet it is a serious psychiatric comorbidity in PLWH. Even mild depression could have deleterious consequences on the lives of PLWH. It can lead to inconsistent adherence, poor care engagement and ultimately to more serious outcomes [42]. Screening for depression among these people should be an integral part of the HIV care package so that it is appropriately managed. The untreated mental disorders work against the successful treatment of PLWH which has a great impact on health outcomes that affect the quality of life. It would also be important to study depression and neurocognitive disorders more rigorously comparing the HIV+ and HIV- cohorts. These screening tools required extensive staff training and time to be administered which was a limitation because the study was conducted within the daily activities of routine HIV care.

## Conclusion

We found that depression was strongly associated with HAND which greatly affects the functioning of an individual. Our findings point to the importance of early recognition of depression and HAND as they are associated with increased rates of poor drug adherence and other poor outcomes including increased risky behaviours.

## Data Availability

The datasets used and/or analyzed during the current study are available from the corresponding author on reasonable request.

## Abbreviations

4WR: Four word recall
AHS: Alternating hand sequence
AIDS: Acquired immune deficiency syndrome
ANOVA: Analysis of variance
ART: Antiretroviral therapy
BDI: Beck depression inventory
CDDP: Community drug distribution point
CNS: Central nervous system
DSM-5: Diagnostic and statistical manual of mental disorders, fifth edition
FT: Finger tapping
HAND: HIV associated neurocognitive disorder
HIV: Human immunodeficiency virus
ICD: International statistical classification of diseases and related health problems
IHDS: International HIV dementia scale
MUST: Mbarara University of Science and Technology
NNRTI: Non-nucleoside reverse transcriptase inhibitor
PI: Principal investigator
PLWH: People living with HIV
PMTCT: Prevention of mother to child transmission
RA: Research assistant
REC: Research ethics committee
SSA: Sub Saharan Africa
TASO: The AIDS support organization
UNAIDS: United Nations agency for international development
UNCST: Uganda National council for science and technology
VCT: Voluntary counseling and testing
